# Sequence Variation in the DDAH1 Gene Predisposes for Delayed Cerebral Ischemia in Subarachnoidal Hemorrhage

**DOI:** 10.3390/jcm9123900

**Published:** 2020-12-01

**Authors:** Juliane Hannemann, Daniel Appel, Miriam Seeberger-Steinmeister, Tabea Brüning, Julia Zummack, Rainer Böger

**Affiliations:** 1 Institute of Clinical Pharmacology and Toxicology, University Medical Center Hamburg-Eppendorf, 20246 Hamburg, Germany; seeberger-steinmeister@web.de (M.S.-S.); t.bruening@asklepios.com (T.B.); julia.zummack@web.de (J.Z.); boeger@uke.de (R.B.); 2 Department of Anesthesiology, University Medical Center Hamburg-Eppendorf, 20246 Hamburg, Germany; d.appel@uke.de

**Keywords:** nitric oxide, genotype-phenotype association, single nucleotide polymorphisms, cerebrovascular disorders, subarachnoidal hemorrhage

## Abstract

Delayed cerebral ischemia (DCI) often causes poor long-term neurological outcome after subarachnoidal hemorrhage (SAH). Asymmetric dimethylarginine (ADMA) inhibits nitric oxide synthase (NOS) and is associated with DCI after SAH. We studied single nucleotide polymorphisms (SNPs) in the NOS3, DDAH1, DDAH2, PRMT1, and AGXT2 genes that are part of the L-arginine–ADMA–NO pathway, and their association with DCI. We measured L-arginine, ADMA and symmetric dimethylarginine (SDMA) in plasma and cerebrospinal fluid (CSF) of 51 SAH patients at admission; follow-up was until 30 days post-discharge. The primary outcome was the incidence of DCI, defined as new infarctions on cranial computed tomography, which occurred in 18 of 51 patients. Clinical scores did not significantly differ in patients with or without DCI. However, DCI patients had higher plasma ADMA and SDMA levels and higher CSF SDMA levels at admission. DDAH1 SNPs were associated with plasma ADMA, whilst AGXT2 SNPs were associated with plasma SDMA. Carriers of the minor allele of DDAH1 rs233112 had a significantly increased relative risk of DCI (Relative Risk = 2.61 (1.25–5.43), *p* = 0.002). We conclude that the DDAH1 gene is associated with ADMA concentration and the incidence of DCI in SAH patients, suggesting a pathophysiological link between gene, biomarker, and clinical outcome in patients with SAH.

## 1. Introduction

Subarachnoidal hemorrhage (SAH) is a major neurosurgical emergency. Vasospasm and cerebral ischemia often occur with some delay during intensive care unit (ICU) treatment of SAH; delayed cerebral ischemia (DCI) is associated with poor clinical outcome [1]. Despite clinical scores and advanced imaging techniques, identification of patients who will develop DCI or not has remained difficult [2].

The L-arginine–nitric oxide (NO) pathway is a major regulator of cerebral vascular tone [3]. NO-mediated cerebral vasodilation is impaired in SAH, thereby eliciting vasospasm and, possibly, DCI [4]. Multiple mechanisms contribute to this reduction in NO. Cell-free hemoglobin in the subarachnoidal space has been shown to scavenge NO [5]; removal of hemoglobin by haptoglobin prevents hemoglobin-induced cerebral vasospasm [6]. Various strategies to restore pulmonary NO levels, e.g., the use of inhaled NO [7], exogenous NO donors, or phosphodiesterase-5 (PDE5) inhibitors, have been shown to mitigate the risk of cerebral vasospasm and ischemia after SAH. One experimental study in haptoglobin 2-2 transgenic mice tested the effects of L-citrulline that is converted to L-arginine after absorption, and found improved cerebral perfusion and neurological outcome [8]. Restoring eNOS substrate concentration has been shown to be effective only when this enzyme is blocked by a competitive inhibitor [9]. Asymmetric dimethylarginine (ADMA) competitively inhibits NO synthesis by displacing the substrate, L-arginine, from the enzyme. We demonstrated in a previous study that higher levels of the endogenous inhibitor of NO synthase (NOS), ADMA, and its congener, symmetric dimethylarginine (SDMA), are markers of DCI and poor neurological outcome in SAH patients [10].

Both ADMA and SDMA are formed during posttranslational protein methylation by protein arginine N-methyltransferases (PRMTs) [11]. In many organs including the vasculature, ADMA is the main product of this process; ADMA is cleaved by either of two isoforms of dimethylarginine dimethylaminohydrolase (DDAH1 and DDAH2) [12]. SDMA is not degraded by DDAHs, but both SDMA and ADMA are substrates of alanine glyoxylate aminotransferase-2 (AGXT2) [13,14].

**Figure 1:**
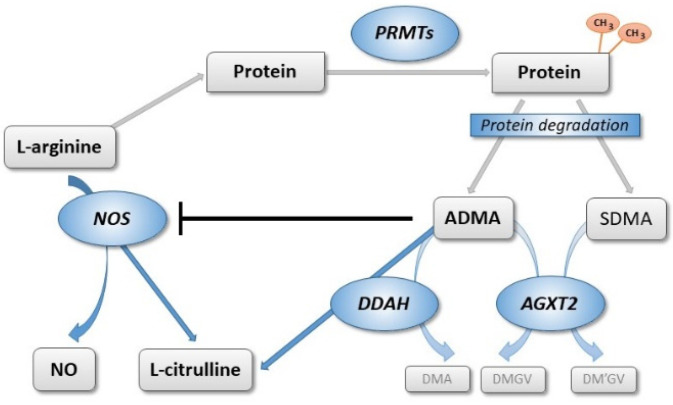
Schematic representation of biosynthesis and metabolism of asymmetric and symmetric dimethylarginine. L-arginine residues within specific proteins are subject to methylation by protein arginine N-methyltransferases (PRMTs). After protein hydrolysis, asymmetric (ADMA) and symmetric dimethylarginine (SDMA) are released. ADMA is a competitive inhibitor of nitric oxide synthases (NOS). ADMA, but not SDMA, is degraded by dimethylarginine dimethylaminohydrolases (DDAH1 and DDAH2) into L-citrulline and dimethylamine (DMA). Both dimethylarginines may be cleaved by an alternative pathway through alanine glyoxylate aminotransferase 2 (AGXT2), resulting in the formation of symmetric or asymmetric dimethylguanidinovaleric acid (DMGV and DM’GV). Figure reproduced with permission from [30] under CC BY-NC (SAGE Publishers).

Figure 1 provides an overview of enzymatic pathways regulating L-arginine and dimethylarginine levels. We have described this pathway and its regulation in detail before [15].

Clinically, ADMA is associated with cardiovascular events and mortality [16,17], whilst SDMA has been associated with mortality after ischemic stroke [18,19]. ADMA is elevated in plasma and/or cerebrospinal fluid (CSF) of patients with SAH [20,21,22] as well as in animal models of SAH [23]. In our previous study [10], we found ADMA and SDMA levels in plasma and CSF of SAH patients to be associated with the incidence of DCI. Therefore, elevated levels of dimethylarginines may result in dysfunction of endothelial NO production, impaired cerebrovascular autoregulation, and vasospasm.

Genotyping studies showed that single nucleotide polymorphisms (SNPs) in the NOS3 gene are associated with an increased incidence of DCI after SAH [24,25] and with poor long-term recovery from SAH [26]. Several single nucleotide polymorphisms (SNPs) in the genes encoding for DDAH1, DDAH2, AGXT2, and PRMT1 have been shown to relate to the plasma concentrations of ADMA or SDMA, respectively, and to be associated with clinical findings of cardiovascular diseases [13,27,28,29]. Based on this data, we hypothesized that genetic variation caused by SNPs in the genes encoding enzymes relating to the L-arginine–dimethylarginine metabolic pathways may be associated with elevated baseline levels of ADMA and SDMA and may contribute to individual susceptibility to DCI after SAH. To test this hypothesis, we genotyped representative SNPs in each of the genes of interest for the L-arginine–dimethylarginine–NO pathway in a cohort of patients admitted with acute SAH.

## 2. Materials and Methods

### 2.1. Study Participants and Protocol

We included 51 consecutive patients who had been admitted to the Intensive Care Unit of the University Medical Center Hamburg-Eppendorf with acute subarachnoidal hemorrhage. The aneurysmal pathogenesis was diagnosed using CT angiography or digital subtraction angiography; in 47 patients, an aneurysm was detected, and in four patients a perimesencephalic subarachnoidal hemorrhage was diagnosed. In 38 patients, external ventricular drain (EVD) insertion was performed on the day of admission because elevation of intracranial pressure was documented. The local ethics committee approved the study protocol (Ethics Committee of the Board of Physicians of Hamburg; PV3622), and written informed consent was obtained from all participants or their next of kin.

The aneurysms were secured by intravascular coiling or surgical clipping as early as possible, at least within 24 h after admission. In eight cases, a non-interventional treatment approach was chosen. Subsequent adjunctive therapies and CT scans were administered when appropriate in adherence with international guidelines defined by the Neurocritical Care Society’s Multidisciplinary Consensus Conference and the American Heart Association (AHA) [31,32]. In accordance with local protocols, oral or intravenous nimodipine was used to prevent cerebral vasospasm in 47 patients. During poor neurological condition or during mechanical ventilation, nimodipine was given intravenously, otherwise orally. Blood gas analysis was performed several times per day, and at least one blood sample per day for routine laboratory parameters was drawn during intensive care therapy, depending on clinical needs. Normoventilation (PaCO_2_ 35–45 mmHg) and normovolemia were controlled closely, and blood pressure was monitored continuously by invasive monitoring. Norepinephrine was primarily used for therapy of episodes of hypotension.

### 2.2. Clinical Assessment and Follow-up

Patients were included on the day of bleeding and were reviewed on days three, six, eight, 12, and 15 after hemorrhage, including a standardized medical history, blood and CSF samples drawn from arterial catheters and external ventricular drains, respectively, and the Glasgow Coma Scale (GCS). CSF samples were only collected from patients with EVD inserted for clinical needs; for ethical reasons, no lumbar punctures were performed solely for study purposes.

At admission, clinical assessment of patients was performed according to standardized procedures including the World Federation of Neurological Surgeons (WFNS) score [33], the GCS [34], and the Hunt and Hess score [35] by board-certified neurosurgeons. Severe clinical condition was defined by a Hunt and Hess score of 3–5. For all patients, the initial CT scan was assessed according to the Fisher classification [36].

The primary outcome parameter was DCI, which was assessed by cranial CT or magnetic resonance imaging performed when signs of neurological impairment became visible to identify new cerebral infarction according to current standards [37]. Transcranial Doppler (TCD) measurements of cerebral arterial flow were taken daily by the same investigator to identify cerebral vasospasm. For statistical analyses, we calculated the maximum increase in mean flow velocity (MFV) related to the day of bleeding (baseline). TCD vasospasm was defined as MFV > 140 cm/s [38].

At discharge, neurological outcome was determined using the Glasgow Outcome Scale (GOS). Poor neurological outcome was defined as GOS < 4. In addition, outcome including the GOS was assessed at 3 months after discharge by patient visits or telephone interviews.

### 2.3. Selection of SNPs

We performed an extensive literature search using PubMed and the National Center for Biotechnology Information (NCBI) SNP database for publications linking SNPs in the DDAH1, DDAH2, ARG1, ARG2, AGXT2, NOS3, and PRMT1 genes to outcome parameters related to the pathophysiology of subarachnoidal hemorrhage, ischemic stroke, and vasospasm. These genes code for the key enzymes in the L-arginine–ADMA/SDMA–NO pathway (Figure 1) [30]. Subsequently, SNPs in the same gene were tested for linkage disequilibrium (LD) using the LDLink database of the National Cancer Institute, USA [39], and the list of SNPs was reduced to the best described SNP for this specific group when strong LD was present; only SNPs that are not in LD were included in the analyses (Table A1).

### 2.4. DNA Isolation and SNP Genotyping

DNA from blood samples was isolated using QIAamp DNA Blood Mini Kit according to the manufacturer’s protocol (Qiagen, Hilden, Germany). DNA was eluted from the columns in a volume of 20 µL. To further purify and concentrate the isolated DNA for SNP genotyping, DNA was precipitated by ethanol precipitation. The DNA pellet was washed once and the resuspended in 20–100 µL H20. DNA concentration was determined using a nanophotometer NP60 (Implen, Munich, Germany).

SNP genotyping was performed using single-tube human TaqMan SNP Genotyping Assays (Thermofisher, Waltham, MS, USA). Each reaction mix contained 10 ng template DNA, 0.5µL specific TaqMan SNP Genotyping Assay and 5 µL of 2xTaqPath ProAmp™ Mastermix in a total reaction volume of 10 µL. The polymerase chain reaction (PCR) was performed in a QuantStudio™ 5 Real-Time PCR System (Thermofisher, Waltham, MS, USA) using the following PCR program: pre-read of 30 s at 60 °C, enzyme activation at 95 °C for 5 min, 40 cycles of denaturation (5 s, 95 °C) and annealing (30 s, 60 °C), followed by a last post-read of 30 s at 60 °C. Allelic calls were identified by Quant Studio Design and Analysis Software (Thermofisher, Waltham, MS, USA).

### 2.5. Biochemical Analyses

At baseline, non-fasting blood and CSF samples were drawn from arterial catheters (N = 51) and external ventricular drains (when available, N = 38), respectively. For ethical reasons, no patient received any interventional measures for study-related sample collection. Samples were centrifuged immediately and stored at −20 °C until analysis. ADMA, SDMA, and L-arginine were determined by liquid chromatography-tandem mass spectrometry (LC–MS/MS) using a previously validated method [40]. In brief, 25 μL of plasma or CSF was diluted with stable isotope-labeled internal standards. Proteins were precipitated with methanol; the guanidine compounds were converted to their butyl esters and analyzed by LC–MS/MS (Varian 1200 MS, Agilent Technologies, Santa Clara, CA, USA). Quantification was performed by calculation of peak area ratios and calibration with known concentrations of analytes in dialyzed EDTA plasma. The analytical range of the method was validated from 0.05 to 4 μmol/L for ADMA and SDMA, respectively, and from 0.5 to 250 μmol/L for L-arginine, and mean coefficients of variation were ≤5% for all analytes. All other laboratory values were measured using routine clinical laboratory methods.

### 2.6. Statistical Analyses

All statistical analyses were performed using SPSS (version 21; IBM Corporation, Armonk, NY, USA). All variables were tested for normal distribution using the Kolmogorov–Smirnov test. Differences between groups were tested for significance by using either the nonparametric Mann–Whitney U test for two groups or the Kruskal–Wallis analysis of variance for more than two groups. Correlations were calculated using linear regression or the Spearman test. Allele frequencies of the genes of interest (GOI) were compared to the European reference population or between groups using contingency tables and Fisher’s exact test. Biomarker levels between genotypes were tested for statistically significant differences by ANOVA followed by the Scheffé f-test, and associations of genotypes with outcome (DCI) were tested for significance by the Chi^2^ test. Haplotypes were constructed for the DDAH1 gene based upon the frequency of homozygosity for the major and minor alleles, respectively. The associations of DDAH1 haplotypes with outcome (DCI) were tested for significance by the Chi^2^ test. Data are presented as median with 25th and 75th percentiles, or as mean with standard error of the mean. For all tests, *p* < 0.05 was considered significant.

## 3. Results

### 3.1. Baseline Characteristics and Clinical Outcome of the Patients

We included in this study 51 patients who were admitted to our Emergency Department with evidence for subarachnoidal hemorrhage. Patients had a mean age of 54.8 ± 13.1 years; 25% of them were men. A total of 43 patients were treated interventionally with either clipping or coiling of the aneurysmal location; 47 patients received nimodipine treatment. The Glasgow Coma Scale was 8.6 ± 5.6 on the day of admission, the WFNS score was 3.4 ± 1.7, and the Hunt and Hess index was 3.1 ± 1.4. Further demographic and anthropometric characteristics of the cohort are listed in Table 1, including risk factor profiles and long-term medical treatment. Thirty-eight patients had an EVD placed on the day of admission for clinical reasons. We found a significantly poorer survival rate in patients with EVD (Relative Risk (RR), 3.897 (95% CI 1.002–15.160), *p* = 0.039), but no significant associations of EVD with the incidence of DCI (RR, 1.271 (95% CI 0.858–1.883), *p* = 0.286) nor vasospasm (RR, 1.542 (95% CI 0.710–3.347), *p* = 0.305).

A total of 18 patients developed DCI during the course of in-patient treatment. At admission, there were no significant differences in any of the demographic and anthropometric parameters between patients who later developed DCI or not (Table 1). Initially, patients who later developed DCI scored even better in the Glasgow Coma Scale than patients who did not (10.9 ± 4.6 vs. 7.2 ± 5.7; *p* = 0.02; Table 2), whilst there were no significant differences in the Hunt and Hess Score, Fisher Score, and WFNS Score (all *p* = n.s.; Table 2). However, the clinical outcome of patients with DCI was more unfavorable than for patients without DCI: There was a trend towards a higher lethality, longer ICU treatment, and longer mechanical ventilation (all *p* = n.s.), as well as a significantly higher rate of re-intervention (*p* = 0.047; Table 2). The Glasgow Outcome Score at discharge and three months later tended to be lower for patients with DCI (2.8 ± 1.6 vs. 3.6 ± 1.5 at discharge, *p* = 0.056; 3.1 ± 1.1 vs. 4.0 ± 1.0 three months later; *p* = 0.071). Vasospasms occurred slightly, but not significantly more frequently in patients with DCI (10 vs. 8 patients) than without DCI (12 vs. 21 patients; *p* = n.s.).

### 3.2. Association of L-Arginine and Dimethylarginines with DCI

**Figure 2:**
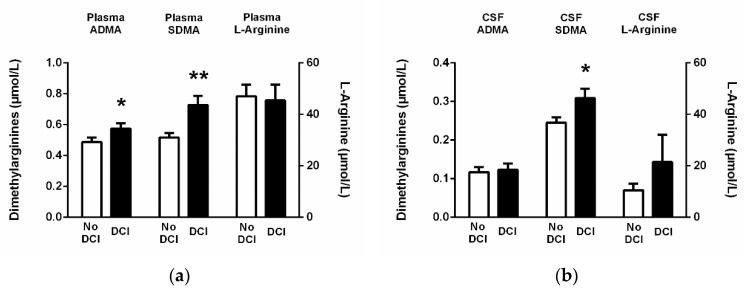
Concentrations of ADMA, SDMA, and L-arginine in (**a**) plasma and (**b**) CSF at the time of admission. A total of 51 patients with SAH were categorized into those who developed delayed cerebral ischemia (N = 18) or not (N = 33). Data are mean ± S.E.M. * *p* < 0.05, ** *p* < 0.01 vs. no DCI group. Abbreviations: ADMA, asymmetric dimethylarginine; CSF, cerebrospinal fluid; DCI, delayed cerebral ischemia; SDMA, symmetric dimethylarginine; S.E.M., standard error of the mean

For L-arginine, there was no significant difference in plasma or CSF between patients with or without DCI. By contrast, the plasma concentrations of ADMA (0.58 ± 0.15 vs. 0.49 ± 0.17 µmol/L; *p* = 0.04) and SDMA at admission (0.73 ± 0.26 vs. 0.52 ± 0.17 µmol/L; *p* = 0.03) were significantly higher in patients who later developed DCI than in those who did not. SDMA concentration was also significantly higher in CSF of patients with DCI than without (0.31 ± 0.10 vs. 0.25 ± 0.08 µmol/L; *p* = 0.03; Figure 2).

### 3.3. Allele Frequencies of Genes of Interest in the Study Cohort Compared to the European Reference Population

By comparison with the European reference population of the 1000 Genomes project, there were significant deviations from the expected distribution of alleles in four of the SNPs that we studied: For NOS3 rs891512, the major allele was more frequent in our cohort, whilst for the DDAH1 rs1241321 and DDAH1 rs480414 SNPs and the DDAH2 rs805304 SNP, the minor allele was more frequent in our cohort (all *p* < 0.05). Table A2 shows allele frequencies of all analyzed SNPs.

### 3.4. Association of SNPs with Biomarkers in Plasma and in Cerebrospinal Fluid

**Figure 3:**
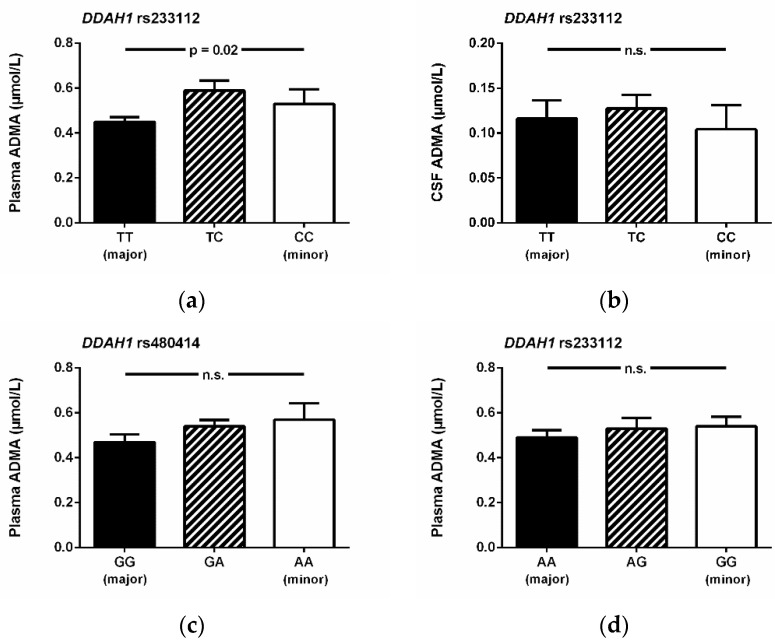
Concentrations of ADMA in plasma or CSF in relation to genotypes of the DDAH1 SNP rs233112 (**a**,**b**), the DDAH1 SNP rs480414 (**c**), and the DDAH1 SNP rs124321 (**d**). Data are mean ± S.E.M. Abbreviations: ADMA, asymmetric dimethylarginine; CSF, cerebrospinal fluid; n.s.; not significant; S.E.M., standard error of the mean; SNP, single nucleotide polymorphism.

**Figure 4:**
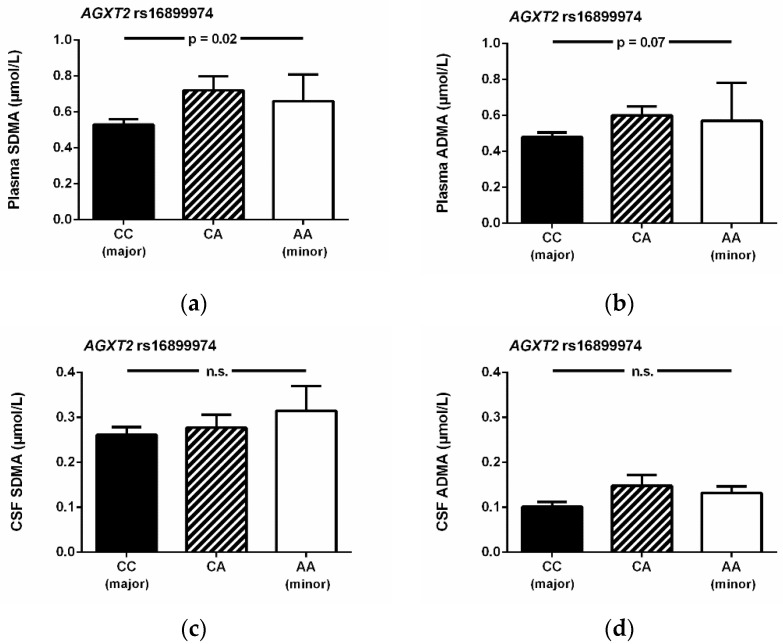
Plasma and CSF concentrations of SDMA (**a**,**c**) and ADMA (**b**,**d**) in relation to genotypes of the AGXT2 rs168999974 SNP. Data are mean ± S.E.M. Abbreviations: ADMA, asymmetric dimethylarginine; CSF, cerebrospinal fluid; SDMA, symmetric dimethylarginine; S.E.M., standard error of the mean; SNP, single nucleotide polymorphism.

There was a significant association of the DDAH1 rs233112 SNP with ADMA plasma concentration, whilst this SNP was not significantly associated with CSF ADMA concentration (Figure 3), nor were any of the other SNPs that we analyzed. By contrast, the DDAH1 SNPs rs480414 and rs1241321 were not significantly associated with ADMA plasma concentration (Figure 3c,d). The AGXT2 SNP rs16899974 was significantly associated with SDMA concentration in plasma but not in CSF (Figure 4a,b) and showed a weak, non-significant association with ADMA concentration in plasma, but not in CSF (Figure 4c,d).

### 3.5. Association of SNPs with the Incidence of Delayed Cerebral Ischemia

**Figure 5:**
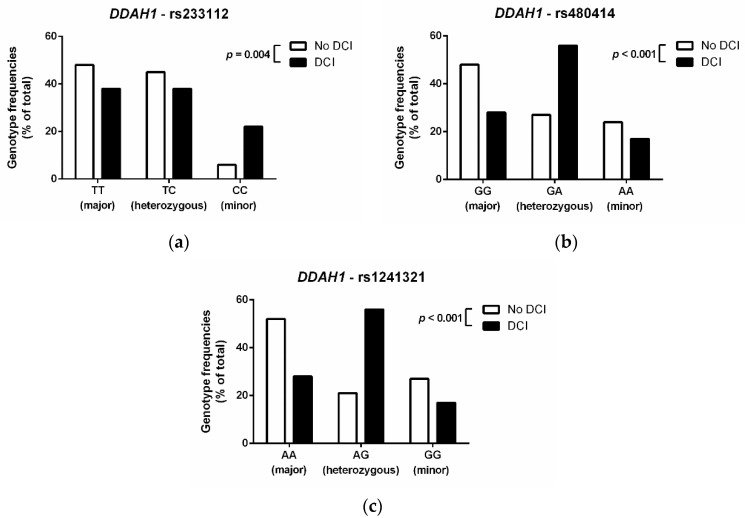
Frequency distribution of genotypes for (**a**) DDAH1 SNP rs233112, (**b**) DDAH1 SNP rs480414, and (**c**) DDAH1 SNP rs1241321 in SAH patients with or without DCI. For all three SNPs, the minor allele was more frequently observed in patients with DCI than in those without (*p* values as indicated in the graphs). Abbreviations: DDAH1, dimethylarginine dimethylaminohydrolase 1; DCI, delayed cerebral ischemia; SAH, subarachnoidal hemorrhage; SNP, single nucleotide polymorphism.

All three SNPs in the DDAH1 gene were significantly associated with the incidence of DCI. In each of the three SNPs, patients with DCI more frequently carried the minor allele than patients without DCI (Figure 5). Patients who were homozygous for the minor allele of DDAH1 rs233112 had a relative risk of 2.61 (95% CI, 1.25–5.43) of developing DCI (*p* = 0.002) as compared to patients who were homozygous for the major allele. By contrast, SNPs in the DDAH2, AGXT2, PRMT1, or NOS3 genes showed no significant associations with DCI.

When we combined genotypes for the three DDAH1 SNPs and analyzed the associations of DDAH1 haplotypes with outcome, we found that as compared to patients homozygous for all three major alleles, individuals homozygous for ≥1 minor allele had a significantly higher relative risk for DCI (RR, 1.316 (95% CI 1.072–1.614), *p* = 0.011) and for poor neurological outcome at ICU discharge (RR, 2.273 (95% CI 1.681–3.072), *p* < 0.0001) and after 3 months (RR, 1.299 (95% CI 1.110–1.519), *p* = 0.001). No significant different was observed for the relative risk of death and vasospasm, respectively (Table 3). Patients who were not homozygous for any of the DDAH1 SNPs had an intermediate risk (Table 3).

## 4. Discussion

Our study has three major findings: Firstly, the plasma concentrations of ADMA and SDMA and the CSF concentration of SDMA at the time of admission are positively associated with the incidence of DCI after subarachnoidal hemorrhage. Secondly, common genetic polymorphisms in the DDAH1 gene are responsible for differences in baseline ADMA plasma concentration, and common genetic polymorphisms in the AGXT2 gene are responsible for differences in plasma SDMA concentration; by contrast, the CSF concentrations of these metabolites were not significantly associated with these SNPs. Thirdly, and most importantly, patients with DCI more frequently carried the minor alleles of any of the three common DDAH1 SNPs that we analyzed; patients homozygous for the minor allele of DDAH1 rs233112 had a significantly higher probability of DCI than patients homozygous for the major allele. Patients carrying the DDAH1 haplotype with the most minor alleles had the highest relative risk for DCI and poor neurological outcome as compared to patients homozygous for the major allele of all three DDAH1 SNPs.

ADMA is an endogenous inhibitor of nitric oxide synthesis. Its plasma levels are positively associated with the incidence of major adverse cardiovascular events and mortality [16]. By inhibiting endothelial NO release [41], high ADMA concentration may cause vasospasm and contribute to ischemia. We have reported a significant association of plasma ADMA with magnetic resonance imaging markers of subclinical vascular brain injury in the Framingham Offspring cohort [42]. These observations are in line with our current results. We have reported before that ADMA plasma concentration increases significantly more in SAH patients developing DCI than in those who do not [10]. However, the initial L-arginine/ADMA ratio (marking the availability of NOS substrate (L-arginine) over NOS inhibitor (ADMA)) at admission was significantly lower in patients who later developed DCI than in those who did not. We therefore hypothesized that genetic predisposition may underlie variable ADMA and SDMA concentrations at admission and be related to outcomes of SAH patients.

In a previous genome-wide association study, we demonstrated that plasma ADMA concentration is associated with the DDAH1 gene, whilst SDMA plasma concentration is associated with the AGXT2 gene in the general population [13]. In our present study, we genotyped common SNPs in genes encoding crucial enzymes of the L-arginine–ADMA–NO pathway, and we confirm in this critically ill population a link between DDAH1 and ADMA plasma concentration and between AGXT2 and SDMA plasma concentration.

Another observation of our study is that the plasma concentrations, but not the CSF levels, of ADMA and SDMA were significantly associated with the DDAH1 and AGXT2 genes, respectively. DDAH1 is highly expressed in liver and kidneys [12] and AGXT2 is primarily located in the renal proximal tubular epithelial cells [43]. These results suggest that ADMA and SDMA circulating in plasma may be cleared by these two enzymatic pathways, whilst dimethylarginines in CSF may not be directly affected by these enzymes. This conclusion is in line with our previous observation that plasma and CSF ADMA concentrations are differentially regulated in Alzheimer’s disease patients [44]. Differential regulation of ADMA in plasma versus CSF was also reported after experimental middle cerebral artery occlusion in rats [45]; ADMA and SDMA concentrations in CSF were found to be elevated immediately after hyperacute ischemic stroke and associated with stroke severity [46].

SDMA does not interfere with NO synthesis directly, but it indirectly modulates NO generation by limiting the intracellular availability of L-arginine via inhibition of the cationic amino acid transporter [47]. Several studies have linked SDMA plasma concentration with ischemic cerebral stroke: In a study of 394 survivors of the first 30 days after acute ischemic stroke, we found that SDMA is associated with total mortality during 7.4 years of follow-up [19]. In another study, we found that cumulative survival during the first 30 days after ischemic stroke decreased significantly with increasing tertiles of plasma SDMA [18]. SDMA is formed during posttranslational methylation of proteins by type II PRMTs [11]; intriguingly, myelin-basic protein, an essential component of myelin sheaths in neuronal tissues, is subject to symmetric dimethylation [48]. Thus, SDMA might be a marker of neurological damage, and in our present study high CSF SDMA levels at admission may point to early neuronal damage in SAH. This observation clearly deserves further investigation.

Beyond its association with ADMA levels, there was a strong association of the DDAH1 gene with outcome after SAH: All three analyzed DDAH1 SNPs were associated with the incidence of DCI, and as compared to a combined genotype (haplotype) containing homozygosity for all three SNPs, haplotypes with an increasing number of minor alleles showed increasingly worse outcome. Thus, functionality of DDAH1 may be an important, in part genetically influenced determinant of the incidence of cerebral ischemia after SAH.

Whilst presenting some exciting observations, our study has limitations. Firstly, the small sample size limits our ability to extrapolate our findings to various levels of severity of SAH. Specifically, the significant deviation of allele distribution in our critically ill cohort from the expected allele frequencies may suggest that genes regulating vascular NO production may predispose for SAH. Clearly, larger cohorts need to be genotyped to confirm and extend our findings. Secondly, due to the strict limitations linked to a clinical study in critically ill patients, we had no opportunity to investigate the mechanisms that may underlie the possible differential regulation of plasma and CSF dimethylarginine levels. This might be better addressed in animal models.

## 5. Conclusions

In conclusion, our study provides evidence that the L-arginine–ADMA–NO metabolic pathway is involved in the pathophysiology of cerebrovascular ischemic complications after subarachnoidal hemorrhage. The DDAH1 gene, probably via determining the metabolism of ADMA, is prominently involved in mediating this risk. Carriers of genetic variants of the DDAH1 gene that lead to malfunction of the enzyme may carry a genetic risk of DCI after SAH.

## Figures and Tables

**Table 1:** Baseline characteristics of the patients.

	All	Patients with DCI	Patients without DCI	*p*
Demographics				
Number of patients	51	18	33	
Age, years (mean ± SD)	54.8 ± 13.1	58.6 ± 14.1	52.6 ± 12.2	0.140
Sex, male/female (%)	13/38 (25.5/75.5)	2/16 (11.2/88.8)	11/22 (33.3/66.7)	0.102
BMI, kg/m^2^ (mean ± SD)	25.9 ± 3.9	26.8 ± 4.9	25.4 ± 3.2	0.333
Risk factors, *n* (%)				
Hypertension	21 (41.2)	9 (50.0)	12 (36.4)	0.385
Diabetes mellitus	3 (5.9)	1 (5.6)	2 (6.1)	1.000
Renal failure	1 (2.0)	1 (5.6)	0 (0.0)	0.353
Smoker, current + ex	11 (21.6)	4 (22.2)	7 (21.2)	1.000
Long-term medication, *n* (%)				
Angiotensin II blockers	6 (11.8)	2 (11.2)	4 (12.1)	0.586
Beta blockers	8 (15.7)	3 (16.7)	5 (15.2)	0.591
Diuretics	3 (5.9)	1 (5.6)	2 (6.1)	0.718
Calcium channel blockers	5 (9.8)	2 (11.2)	3 (9.1)	0.585
Statins	6 (11.8)	2 (11.2)	4 (12.1)	0.646
NSAR	3 (5.9)	1 (5.6)	2 (6.1)	0.718
Platelet inhibitors	5 (9.8)	2 (11.2)	3 (9.1)	0.585
Oral anticoagulants	1 (2.0)	0 (0.0)	1 (3.0)	0.647
Bleeding location, *n* (%)				
Anterior cerebral circulation	35 (64.8)	12 (66.7)	23 (69.7)	0.532
Posterior cerebral circulation	12 (22.2)	4 (22.2)	8 (24.2)	0.579
Aneurysm treatment, *n* (%)				
Clipping	15 (29.4)	6 (33.3)	9 (27.3)	0.442
Coiling	28 (54.9)	9 (50.0)	18 (54.5)	0.492
Non-interventional	8 (15.7)	2 (11.1)	6 (18.2)	0.409
Nimodipin treatment	47 (92.2)	17 (94.4)	30 (90.9)	0.557

Abbreviations: BMI, body mass index; DCI, delayed cerebral ischemia; NSAR, non-steroidal antirheumatic drugs; SD, standard deviation. *p* values are given for differences between patients with and without DCI.

**Table 2:** Clinical scores and outcome of SAH patients with or without DCI.

	All	Patients with DCI	Patients without DCI	*p*
Number of patients	51	18	33	
Scores at admission (mean ± SD)				
GCS	8.6 ± 5.6	10.9 ± 4.6	7.2 ± 5.7	0.021
Hunt and Hess	3.1 ± 1.4	2.9 ± 1.2	3.2 ± 1.5	0.622
Fisher Score	3.3 ± 0.7	3.2 ± 0.8	3.4 ± 0.7	0.416
WFNS	3.4 ± 1.7	2.9 ± 1.5	3.6 ± 1.8	0.134
Outcome, *n* (%)				
Vasospasm	22 (43.1)	10 (55.6)	12 (36.4)	0.242
In-hospital lethality	7 (13.7)	3 (16.7)	4 (12.1)	0.686
Duration of ICU treatment (d)	21.0 ± 11.5	22.7 ± 11.2	20.1 ± 11.7	0.449
Mechanical ventilation (h)	237.5 ± 272.5	313.3 ± 251.7	196.1 ± 278.2	0.144
Re-intervention rate	5 (9.8)	4 (22.2)	1 (3.0)	0.047
GOS at discharge	3.4 ± 1.6	2.8 ± 1.6	3.6 ± 1.5	0.056
GOS three months later	3.6 ± 1.1	3.1 ± 1.1	4.0 ± 1.0	0.071

Abbreviations: GCS, Glasgow Coma Scale; WFNS, World Federation of Neurological Surgeons; GOS, Glasgow Outcome Scale. *p* values are given for differences between patients with and without DCI.

**Table 3:** Association of the combined DDAH1 haplotype with outcome parameters.

DDAH1 Haplotype	Relative Risk	95% CI	*p*
**(3× homozygous major versus 0× homozygous minor)**			
DCI	1.103	0.925–1.315	0.273
Vasospasm	1.483	1.013–2.170	**0.039**
Death	1.000	0.898–1.113	1.000
GOS < 4 at discharge	1.364	1.105–1.683	**0.003**
GOS < 4 after 3 months	1.475	1.231–1.766	**<0.0001**
**(3× homozygous major versus ≥ 1× homozygous minor)**			
DCI	1.316	1.072–1.614	**0.011**
Vasospasm	0.983	0.774–1.247	1.000
Death	0.929	0.460–1.874	1.000
GOS < 4 at discharge	2.273	1.681–3.072	**<0.0001**
GOS < 4 after 3 months	1.299	1.110–1.519	**0.001**

Details of the haplotype construction are given in Appendix A. Abbreviations: DCI, delayed cerebral ischemia; GOS, Glasgow outcome scale. *p* values presented were calculated by Chi^2^ test; significant *p* values are printed in bold type. Abbreviation: 95% CI, 95% confidence interval.

## Appendix A

### Appendix A.1. Selection and Clinical Significance of the Selected Single Nucleotide Polymorphisms

In this study, we aimed to investigate whether genetic variance in the genes encoding for enzymes relating to the L-arginine–dimethylarginine metabolic pathways may be associated with elevated baseline levels of ADMA and SDMA and may contribute to individual susceptibility to DCI after SAH. A number of enzymes metabolize the biomarkers associated with this pathway (see Figure 1 in the main manuscript): Endothelial nitric oxide synthase (eNOS) is the enzyme that generates nitric oxide (NO), the major vasodilator mediator, from L-arginine. Another enzyme that metabolizes L-arginine is arginase. By converting L-arginine into L-ornithine, arginase may reduce the availability of substrate for NO synthesis and thereby indirectly influence NO production. Arginase is present in two isoforms (arginase-1 and arginase-2). NOS is inhibited by methylarginines; NG-monomethyl-L-arginine (L-NMMA), asymmetric and symmetric dimethylarginine (ADMA and SDMA) are present endogenously. They are formed during the process of posttranslational protein methylation by a family of enzymes called protein arginine N-methyltransferases (PRMTs). PRMT1 is the most abundant isoform of these enzymes that is present in many cell types. L-NMMA and ADMA are metabolized by either of two isoforms of dimethylarginine dimethylaminohydrolase (DDAH1 and DDAH2), which are present in different tissues and apparently underlie different mechanisms of regulation. ADMA and SDMA, in turn, are both substrates of alanine glyoxylate aminotransferase-2 (AGXT2). We have described this pathway and its regulation in detail before [15].

Genetic variability studies on the corresponding genes have been performed previously by numerous groups of investigators. Table A1 gives an overview of the SNPs that were selected for this study, including references to previous studies linking variability to outcome parameters related to the pathophysiology of subarachnoidal hemorrhage, ischemic stroke, and vasospasm. For some of the SNPs (e.g., in the NOS3, DDAH1, DDAH2, and AGXT2 genes), we made a selection of references relating the SNP to clinical parameters linked to the topic of our present study; for some other SNPs, there was comparatively little published information available (e.g., in the ARG1, ARG2, and PRMT1 genes). For the NOS3 gene, a vast number of different SNPs have been described; however, many of these are in linkage disequilibrium (LD) based on information available in the LDLink database of the National Cancer Institute, USA [39], so we selected one tag SNP out of a specific group. For ARG1, ARG2, and PRMT1, we used the same strategy of testing for LD in order to identify SNPs spread over the whole gene even if no clinical associations have been published yet.

### Appendix A.2. Construction of DDAH1 Haplotypes

To assess the association of the DDAH1 gene with clinical outcome after SAH beyond the effects of individual SNPs in this gene, we constructed haplotypes based upon the numbers of homozygosities in these three gene loci. Haplotype 1 was defined as homozygous presence of the major allele for all three loci; this haplotype was used as reference in the statistical analyses (*n* = 8). Haplotype 2 was an intermediate haplotype, comprising individuals who were homozygous for the minor allele in none of the three DDAH1 loci and not simultaneously homozygous for the major alleles of all three loci (*n* = 22). Haplotype 3 comprised individuals who carried at least one homozygosity for the minor allele in any of the loci (*n* = 21). This classification allowed us to study the gene dose effect of DDAH1 on outcome, with the frequency of the major alleles gradually decreasing from haplotype 1 to haplotype 3. The associations of haplotypes with the following clinical outcome measures were assessed by Chi^2^ test: incident DCI, death, incident cerebral vasospasm, GOS at discharge, GOS at three months after hospital discharge.

**Table A1:** Summary of SNPs selected for this study and their previously published associations with clinical parameters.

Gene/SNP	Position	Genetic Consequence	Clinical Rationale	Related References
NOS3				
rs1799983	Exon 8	Missense variant	Minor allele associated with worse outcome after SAH and risk of ischemic stroke.	[26,49,50,51,52]
rs2070744	Intron 1	Intron variant	Minor allele associated with increased risk of DCI and cardiac instability in patients with SAH; proposed as a susceptibility locus for cerebral infarction.	[53,54,55]
rs891512	Intron 23	Intron variant	Minor allele associated with ankle-brachial index, with higher systolic and diastolic blood pressure, and risk of coronary artery disease.	[56,57,58,59,60]
DDAH1				
rs1241321	Intron 1	Intron variant	Minor allele associated with higher risk of all-cause mortality and the combined endpoint of cardiovascular death, nonfatal myocardial infarction and stroke.	[61]
rs233112	Exon 6	3′ UTR variant	Minor allele associated with arterial stiffness and pulse wave reflection.	[62]
rs480414	Intron 1	Intron variant	Minor allele associated with a lower incidence of pulmonary hypertension in patients with bronchopulmonary dysplasia.	[63]
DDAH2				
rs805304		2 kb upstream variant	Minor allele associated with higher prevalence of hypertension, higher ADMA concentration in diabetic renal impairment, and decreased risk of myocardial infarction.	[64,65,66]
rs2272592		2 kb upstream variant	Minor allele associated with type 2 diabetes.	[67]
ARG1				
rs2246012	Intron 2	Intron variant	No published information available.	
rs2781667	Intron 1	Intron variant	Major allele associated with higher severity of erectile dysfunction.	[68]
ARG2				
rs3742879	Intron 7	Intron variant	Major allele associated with lower exhaled nitric oxide in children, and more severe airway obstruction in patients with asthma.	[69,70]
rs3759757		8 kb upstream variant	No published information available.	
AGXT2				
rs37369	Exon 4	Missense variant	Minor allele associated with higher methylarginine levels, higher diastolic blood pressure, and higher risk of coronary artery disease.	[71,72,73]
rs16899974	Exon 14	Missense variant	Minor allele associated with higher levels of SDMA, with atrial fibrillation and ischemic stroke, and higher risk of coronary artery disease.	[71,73,74]
PRMT1				
rs10415880	Intron 8	Intron variant	Minor allele associated with arteriovenous fistula malfunction in male hemodialysis patients.	[75]
rs975484		Upstream transcript variant	No published information available.	

Abbreviations: NOS3, endothelial nitric oxide synthase; DDAH1, dimethylarginine dimethylaminohydrolase-1; DDAH2, dimethylarginine dimethylaminohydrolase-2; ARG1, arginase-1; ARG2, arginase-2; AGXT2, alanine glyoxylate aminotransferase-2; PRMT1, protein-arginine N-methyltransferase-1.

## Appendix B

**Table A2:** Allele frequencies of studied SNPs.

Gene/SNP	Major/Minor Allele	Allele Frequency Measured	Allele Frequency Expected ^#^	*p*
NOS3				
rs1799983	G/T	0.706/0.294	0.656/0.344	0.223
rs2070744	T/C	0.598/0.402	0.562/0.438	0.530
**rs891512**	**G/A**	**0.843/0.157**	**0.751/0.249**	**0.039**
DDAH1				
**rs1241321**	**A/G**	**0.598/0.402**	**0.712/0.288**	**0.023**
rs233112	T/C	0.667/0.333	0.617/0.383	0.338
**rs480414**	**G/A**	**0.598/0.402**	**0.698/0.302**	**0.043**
DDAH2				
**rs805304**	**T/G**	**0.529/0.471**	**0.639/0.361**	**0.032**
rs2272592	C/T	0.892/0.108	0.844/0.156	0.246
ARG1				
rs2246012	C/T	0.853/0.147	0.680/0.320	0.677
rs2781667	T/C	0.676/0.324	0.829/0.171	1.000
ARG2				
rs3742879	G/C	0.696/0.304	0.741/0.259	0.911
rs3759757	A/G	0.755/0.245	0.687/0.313	0.813
AGXT2				
rs37369	C/A	0.075/0.925	0.773/0.227	1.000
rs16899974	C/T	0.185/0.815	0.913/0.086	0.385
PRMT1				
rs10415880	G/A	0.407/0.593	0.738/0.262	0.158
rs975484	C/G	0.685/0.315	0.658/0.342	0.197

# Expected allele frequencies were calculated using the allele distributions in the European population of the 1000 Genomes Project (N = 1006) [76]. The *p* values denote the level of statistical significance for the comparison of absolute frequencies of the minor and major allele in Fisher’s exact test (two-sided). Alleles for which the distribution significantly between the study population and the European reference population are printed in bold type. A *p* < 0.05 was considered significant. Abbreviations: NOS3, endothelial nitric oxide synthase; DDAH1, dimethylarginine dimethylaminohydrolase-1; DDAH2, dimethylarginine dimethylaminohydrolase-2; ARG1, arginase-1; ARG2, arginase-2; AGXT2, alanine glyoxylate aminotransferase-2; PRMT1, protein-arginine N-methyltransferase-1.
